# Effect of erythropoiesis-stimulating agent types on malignancy in hemodialysis patients

**DOI:** 10.1093/ckj/sfaf148

**Published:** 2025-06-16

**Authors:** Seok Hui Kang, Yu Jeong Lim, Bo Yeon Kim, Ji Young Choi, Jun Young Do

**Affiliations:** Division of Nephrology, Department of Internal Medicine, College of Medicine, Yeungnam University, Daegu, Republic of Korea; Health Insurance Review and Assessment Service, Wonju, Republic of Korea; Health Insurance Review and Assessment Service, Wonju, Republic of Korea; Health Insurance Review and Assessment Service, Wonju, Republic of Korea; Division of Nephrology, Department of Internal Medicine, College of Medicine, Yeungnam University, Daegu, Republic of Korea

**Keywords:** erythropoiesis-stimulating agent, hemodialysis, malignancy

## Abstract

**Background:**

Since erythropoiesis-stimulating agent (ESA) types vary in their affinity for receptors, investigating their association with malignancies could offer valuable insights. This study aims to evaluate the effect of ESA types on malignancy incidence in hemodialysis (HD) patients.

**Methods:**

The Health Insurance Review and Assessment Service has operated a nationwide HD quality assessment program to address the high medical costs and mortality rates among HD patients. This retrospective study analyzed data from 33 960 HD patients, who underwent fourth and fifth HD quality assessments. Participants were divided into three groups: short-, intermediate- and long-acting groups. The onset of any malignancy was defined as the date of the first diagnosis based on International Classification of Diseases, Tenth Revision codes for the 12 most common malignancies. Patient survival was assessed for those with a first diagnosis of any malignancy during follow-up.

**Results:**

The short-, intermediate- and long-acting groups comprised 26 006, 6448 and 1506 patients, respectively (over ∼75 months of follow-up). The 5-year malignancy-free rates were 88.4%, 88.2% and 87.0% for short-, intermediate- and long-acting groups, respectively (*P* = .024 for short/intermediate-acting vs long-acting group). Univariable and multivariable analyses showed higher malignancy risk in the long-acting group, especially in males, older individuals and those on higher ESA doses. We performed analyses using a balanced cohort after propensity score weighting. The balanced cohort also confirmed higher malignancy risk in the long-acting group, while survival rates remained unaffected by ESA type.

**Conclusion:**

Our population-based cohort study reveals an association between long-acting ESAs use and the incidence of any malignancy, with a particularly strong influence on high-dose users. This suggests that avoiding long-acting ESAs may be advisable for patients at high risk of malignancy.

KEY LEARNING POINTS **What was known:**

- The use of erythropoiesis-stimulating agents (ESAs) to manage anemia in hemodialysis (HD) patients has been associated with a higher incidence of malignancies and the progression of existing cancers.
- However, studies exploring the relationship between ESA types (short-, intermediate- or long-acting) and malignancies in HD patients are limited.

**This study adds:**

- The use of long-acting ESA increased the risk of any malignancy by 21% in the short-acting ESA group and 17% in the intermediate-acting ESA group.
- Subgroup analysis revealed that, among patients administered high doses of ESA, the incidence of any malignancy was significantly higher in those using long-acting ESA than in those using other ESA formulations.

**Potential impact:**

- Our study reveals an association between long-acting ESAs use and the incidence of any malignancy, with a particularly strong influence on high-dose users.
- This suggests that avoiding long-acting ESAs may be advisable for patients at high risk of malignancy.

## INTRODUCTION

Malignancy has recently emerged as a major chronic complication affecting the prognosis of hemodialysis (HD) patients. Butler *et al*. show that the incidence of all-site malignancies in HD patients is 1.4 times higher than that in the general population [1]. However, the dialysis-specific mechanisms underlying this risk remain unclear, partly due to confounding factors such as cardiovascular mortality. Recent studies show that factors such as age, longer dialysis duration, sex, non-diabetic status, immune dysfunction, uremia, chronic inflammation, viral infections, increased DNA damage with impaired repair capacity, carcinogen accumulation and exposure to immunosuppressive agents may contribute to cancer development in this population [2, 3]. However, given the complex nature of cancer types as well as regional and national differences, further research is needed to comprehensively understand the cancer risk factors in patients undergoing dialysis.

Anemia is prevalent in patients undergoing HD, and the use of erythropoiesis-stimulating agents (ESAs) is essential for its management. Although earlier studies link ESA use in patients with pre-existing cancer to poor survival outcomes and cancer progression, more recent studies show inconsistent results, suggesting no significant association between ESA use and adverse cancer outcomes [4–6]. Given these inconsistent findings, the American Society of Clinical Oncology/American Society of Hematology guidelines recommend caution in using ESA for patients with active cancer [7]. Given the high prevalence of anemia in HD patients, the widespread use of ESAs as a standard treatment and the higher cancer burden in HD patients than in the general population, investigating the association between ESA use and malignancy in HD patients remains an important area of research.

Previous studies have linked ESA doses or use to malignancy incidence [4–7]. However, studies exploring the relationship between ESA types (short-, intermediate- or long-acting) and malignancies in HD patients are limited. The risk of malignancy associated with ESA use is thought to stem from the activation of erythropoietin receptors [8]. Since ESA types vary in their affinity for erythropoietin receptors, investigating their association with malignancies could offer valuable insights [9]. Therefore, this study aims to evaluate the effect of ESA types on malignancy incidence in maintenance HD patients using a population-based cohort.

## MATERIALS AND METHODS

### Data sources and study population

In our retrospective study, datasets from patients who underwent periodic HD quality assessments and their claims data were analyzed [10, 11]. In South Korea, the Health Insurance Review and Assessment Service (HIRA) operates a nationwide HD quality assessment program to address the high medical costs and mortality rates in HD patients [11]. This program regularly evaluates the status of HD centers and provides differential incentives based on the quality of care. The HD quality assessment program collects patient demographics, along with key laboratory and clinical parameters associated with outcomes in HD patients, over a 6-month assessment period. The first HD quality assessment program was performed between October and December 2010. Data from the fourth (July and December 2013) and fifth (July and December 2015) HD quality assessment programs were used in this study. These programs included adult patients (≥18 years) who had undergone maintenance HD (≥3 months and two or more times per week). Data from the relevant HD quality assessment and claims data for all patients were analyzed. The fourth and fifth assessments included approximately 41.7% and 56.7% of all prevalent patients with HD in South Korea, respectively [12].

Among 57 335 patients included in the fourth and fifth assessments, we excluded the following groups: repeat participants (*n* = 13 789), patients undergoing HD through a catheter (*n* = 958), those with insufficient data (*n* = 181), those without an ESA prescription during the assessment (*n* = 5233), those with International Classification of Diseases, Tenth Revision (ICD-10) codes for malignancy in the year before or within 6 months of the assessment (*n* = 2991) and those who received transfusions during the 6-month assessment period (*n* = 223). Overall, 33 960 patients were included in this study. This study was approved by the institutional review board of Yeungnam University Medical Center (approval no. YUMC 2023-12-012). Informed consent was not required, as patient records and information were anonymized and de-identified before analysis. The institutional review board of Yeungnam University Medical Center waived the informed consent requirement owing to the retrospective nature of the study. This research was conducted in adherence to ethical standards outlined in the Declaration of Helsinki.

### Study variables

During each HD quality assessment, data were collected on age, sex and vascular access type. Additional clinical measures included hemoglobin (g/dL), body mass index (kg/m^2^), HD vintage, Kt/V_urea_, serum albumin (g/dL), serum calcium (mg/dL), serum phosphorus (mg/dL), serum creatinine (mg/dL), transferrin saturation (%), ferritin (ng/mL) and ultrafiltration volume (L/session). These data were collected monthly, with all laboratory values averaged from the monthly recordings. Kt/V_urea_ was calculated using the Daugirdas equation [13]. ESA dose (IU/week) was averaged over 6-month period. The erythropoietin resistance index (ERI) was calculated using the following equation: ERI = ESA dose (IU/week)/body weight (kg)/hemoglobin level (g/dL) [14].

Supplementary data, Table S1 presents the medication codes. Participants were divided into the following groups based on the ESA type used during the 6-month assessment period: short-, intermediate- and long-acting groups. Patients using epoetin-alfa or epoetin-beta were placed in the short-acting group. Those using darbepoetin-alfa were placed in the intermediate-acting group. Patients using continuous erythropoietin receptor activators were classified into in the long-acting group. Patients who used two or more ESAs were assigned to the ESA group corresponding to the highest doses taken during the 6 months. The doses of the various ESAs were converted to a uniform unit (IU/week) using a conversion ratio from a previous study [15]. Various studies report ESA conversion rates that are generally consistent, but some differences exist. Most of these standards are derived from manufacturer-established conversion ratios approved by the Food and Drug Administration (FDA). In our study, we applied FDA-approved conversion standards [16, 17], using a 200:1 ratio for epoetin-alfa or -beta to darbepoetin-alfa and manufacturer-recommended guidelines for continuous erythropoietin receptor activator. Although recent studies report that the epoetin-to-darbepoetin conversion ratio may exceed 200:1, prompting recommendations for adjustments, many large-scale epidemiological studies continue to use the same or similar standards to those applied in our study [18, 19].

Medications, including renin–angiotensin system blocker (RASB), aspirin, clopidogrel and statins were evaluated. Medication use was defined as one or more prescription identified during the HD quality assessment program. Comorbidities were assessed for 1 year before the HD quality assessment. The Charlson comorbidity index (CCI) was used to define comorbidities, which included 17 comorbidities. CCI scores were calculated for all patients [20, 21]. Additionally, myocardial infarction (MI) or congestive heart failure (CHF) were identified using ICD-10 codes.

Outcomes were evaluated from the endpoint of each HD quality assessment program to the follow-up endpoint (June 2024). The onset of any malignancy was defined as the date of the first diagnosis based on ICD-10 codes for the 12 most common malignancies in South Korea [22]. These malignancies include thyroid (C73), lung (C33, C34), colorectal (C18-21), stomach (C16), breast (C50), prostate (C61), liver (C22), pancreas (C25), gallbladder or biliary duct (C23, C24), kidney (C64, C65), uterus or cervix (C53-55), and bladder (C67). In our study, incidence or mortality data were evaluated at the endpoint of each HD quality assessment program. The follow-up began on 1 January 2014, for patients in the fourth program and 1 January 2016, for patients in the fifth program. The malignancy-free interval was calculated from the endpoint of each HD quality assessment program to the first diagnosis of any malignancy, death or censoring. Patient survival assessed for those with a first diagnosis of any malignancy during follow-up. Survival duration was calculated from the first diagnosis of malignancy to death or censoring. Data on peritoneal dialysis or kidney transplantation were censored at this point.

### Statistical analyses

Data were analyzed using SAS Enterprise Guide v.7.1 and R v.3.5.1. Categorical variables were presented as frequencies and percentages, while continuous variables were expressed as means with standard deviations. Statistical significant differences between categorical variables were assessed using Pearson's χ^2^ test or Fisher's exact test. Differences between continuous variables were examined using a one-way analysis of variance with Tukey's *post hoc* test. Survival estimates were calculated using Kaplan–Meier curves and Cox regression analyses. The log-rank test was used to determine the *P*-values for comparing survival curves.

Hazard ratios (HRs) and confidence intervals were calculated using Cox regression analyses. Multivariable Cox regression analyses were adjusted for the following variables: age, sex, body mass index, vascular access type, diabetes, HD vintage, CCI score, ultrafiltration volume, Kt/V_urea_, hemoglobin, serum albumin, serum creatinine, serum phosphorus and serum calcium levels; use of RASB, statins, clopidogrel or aspirin; presence of MI or CHF; ESA dose per week; ERI; transferrin saturation rate; and ferritin levels. These analyses were performed using the enter mode. Subgroup analyses were based on median age, sex and median ESA dose (<5660 IU/week for the low group and ≥5660 IU/week for the high group). Statistical significance was determined at *P* < .05.

Significant differences in baseline characteristics were observed among the three groups. To mitigate potential bias, we applied propensity score weighting to balance these characteristics, ensuring the validity of our analyses. We constructed balanced cohort for the three groups using generalized boosted models, adjusting for the following variables: age, sex, body mass index, diabetes status, vascular access type, CCI score, HD vintage, ultrafiltration volume, Kt/V_urea_, hemoglobin, creatinine, phosphorus, albumin, calcium, transferrin saturation, ferritin; ERI levels; ESA dose; the administration of aspirin, statin, RASB or clopidogrel; and the presence of MI or CHF. Propensity scores were used to calculate inverse probability treatment weights. The balanced cohort was defined as a sample with weights assigned to each case, with continuous variables presented as means and standard errors. *P*-values were tested using a general linear model with a complex survey design, incorporating sample weights.

## RESULTS

### Baseline characteristics

The short-, intermediate- and long-acting groups comprised 26 006, 6448 and 1506 patients, respectively (Table 1). The patients in the short-acting group were younger than those in the other two groups. Additionally, this group had higher HD vintage, ultrafiltration volume, serum albumin, calcium and creatinine levels than in the other groups. The short-acting group had higher proportion of males and clopidogrel use than those in the other two groups. However, this group exhibited lower body mass index, Kt/V_urea_ and hemoglobin levels, as well as a lower proportion of individuals using RASB or statins, or having history of MI or CHF, than those in the other two groups. The long-acting group exhibited higher CCI scores and transferrin saturation rates than those of the other two groups. The follow-up durations for the short-, intermediate- and long-acting groups were 74 ± 39, 73 ± 39 and 76 ± 38 months, respectively.

**Table 1:** Baseline characteristics.

	Short-acting (*n* = 26 006)	Intermediate-acting (*n* = 6448)	Long-acting (*n* = 1506)	*P*-value
Age (years)	60.1 ± 12.8	60.9 ± 12.9^a^	61.5 ± 12.6^a^	<.001
Sex (male), *n* (%)	15 106 (58.1)	3578 (55.5)	864 (57.4)	<.001
HD vintage (months)	63 ± 64	56 ± 57^a^	55 ± 59^a^	<.001
Body mass index (kg/m^2^)	22.3 ± 3.3	22.5 ± 3.5^a^	22.7 ± 3.2^a^,^b^	<.001
Diabetes, *n* (%)	11 399 (43.8)	2991 (46.4)	699 (46.4)	<.001
CCI score	6.6 ± 2.6	6.6 ± 2.5	6.9 ± 2.6^a^,^b^	<.001
Arteriovenous fistula, *n* (%)	22 238 (85.5)	5453 (84.6)	1283 (85.2)	.159
Kt/V_urea_	1.52 ± 0.27	1.56 ± 0.29^a^	1.56 ± 0.27^a^	<.001
Ultrafiltration volume (L/session)	2.31 ± 0.96	2.26 ± 1.00^a^	2.22 ± 1.01^a^	<.001
Hemoglobin (g/dL)	10.6 ± 0.7	10.7 ± 0.7^a^	10.7 ± 0.8^a^	<.001
Serum albumin (g/dL)	4.00 ± 0.34	3.93 ± 0.34^a^	3.95 ± 0.31^a^	<.001
Serum phosphorus (mg/dL)	4.99 ± 1.34	4.82 ± 1.28^a^	4.96 ± 1.38^b^	<.001
Serum calcium (mg/dL)	8.94 ± 0.85	8.88 ± 0.72^a^,^b^	8.86 ± 0.81^a^	<.001
Serum creatinine (mg/dL)	9.6 ± 2.7	9.4 ± 2.7^a^	9.1 ± 2.6^a^,^b^	<.001
Use of RASB, *n* (%)	17 972 (69.1)	4657 (72.2)	1063 (70.6)	<.001
Use of aspirin, *n* (%)	3450 (13.3)	697 (10.8)	208 (13.8)	<.001
Use of clopidogrel, *n* (%)	2131 (8.2)	372 (5.8)	112 (7.4)	<.001
Use of statins, *n* (%)	10 531 (40.5)	2951 (45.8)	671 (44.6)	<.001
MI or CHF, *n* (%)	10 153 (39.0)	2660 (41.3)	604 (40.1)	.004
Transferrin saturation (%)	32.7 ± 14.0	33.1 ± 14.9	36.0 ± 14.3^a^,^b^	<.001
Ferritin	262 ± 259	287 ± 288^a^,^b^	269 ± 242^b^	<.001
ERI [(IU/week)/kg/(g/dL)]	10.5 ± 6.5	9.7 ± 7.1^a^	6.1 ± 3.9^a^,^b^	<.001

Data are expressed as means ± standard deviation for continuous variables and as numbers (percentages) for categorical variables.

*P*-values were tested using one-way analysis of variance, followed by the Tukey *post hoc* test. Pearson's χ^2^ test was performed for categorical variables.

^a^ *P* < .05 vs short acting; ^b^*P* < .05 vs intermediate acting.

We divided the study population into two cohorts based on the HD quality assessment program and compared their baseline characteristics. The number of patients in the short-, intermediate- and long-acting groups was 11 853 (77.9%), 2758 (18.1%) and 603 (4.0%) in the fourth program, and 14 153 (75.5%), 3691 (19.7%) and 903 (4.8%), in the fifth program. Supplementary data, Table S2 shows the baseline characteristics based on different programs. Significant differences were observed in most baseline characteristics across ESA types, with trends consistent with those in the total cohort. In the fourth program, the follow-up durations for those with short-, intermediate- and long-acting groups were 77 ± 43, 74 ± 44 and 79 ± 43 months, respectively. In the fifth program, the corresponding durations were 73 ± 35, 71 ± 35 and 73 ± 34 months. Although the follow-up duration was longer in the fourth program than that in the fifth program, the difference was minimal.

### Any malignancy or mortality according to groups

**Figure 1:**
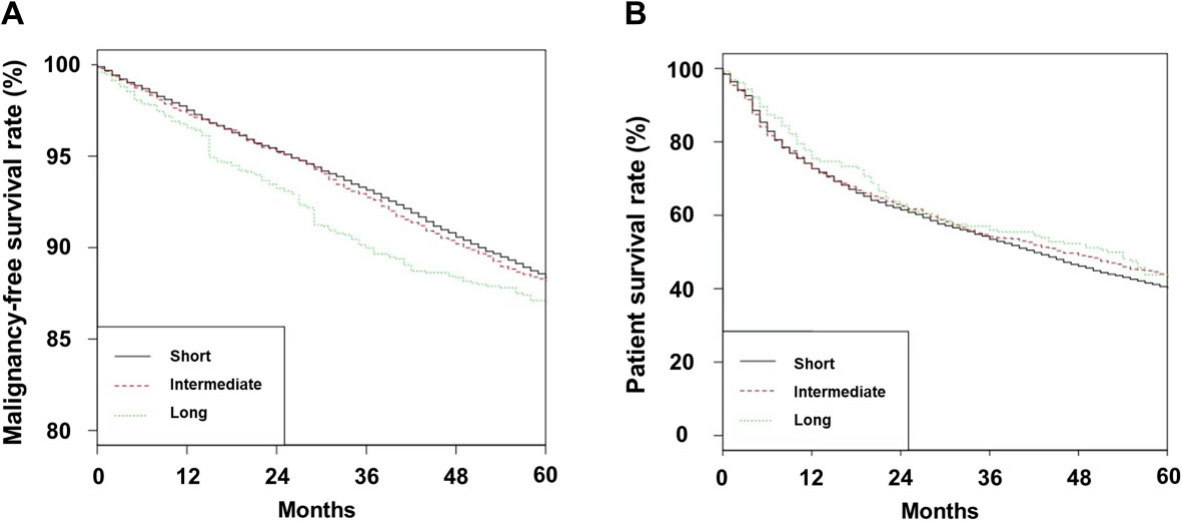
Kaplan–Meier curves by type of ESAs. (**A**) Malignancy-free survival and (**B**) patient survival rates.

The 5-year malignancy-free rates were 88.4%, 88.2% and 87.0% for short-, intermediate- and long-acting groups, respectively (Fig. 1A; *P* = .060 for trends, *P* = .024 for short/intermediate-acting vs long-acting group). The 5-year patient survival rates were 40.3%, 43.2%, and 43.1% for the short-, intermediate- and long-acting groups, respectively (Fig. 1B; *P* = .400 for trends, *P* = .450 for short/intermediate-acting vs long-acting group).

Univariable analysis showed that the HR for any malignancy was higher in the long-acting group than in the short-acting group (Table 2). Multivariable analysis confirmed that the HR was higher in the long-acting group than in the short- and intermediate-acting groups. However, patient survival in those with any malignancy was not associated with the type of ESA agents used.

**Table 2:** ESA types and HR of malignancy or patient survival.

	Univariable		Multivariable	
	HR (95% CI)	*P*-value	HR (95% CI)	*P*-value
Malignancy				
Ref: Short-acting group				
Intermediate-acting group	1.02 (0.95–1.10)	.531	1.03 (0.95–1.12)	.453
Long-acting group	1.17 (1.02–1.33)	.021	1.21 (1.05–1.39)	.007
Ref: Intermediate-acting group				
Long-acting group	1.14 (0.99–1.31)	.073	1.17 (1.01–1.36)	.038
Mortality				
Ref: Short-acting group				
Intermediate-acting group	0.95 (0.86–1.05)	.284	0.92 (0.83–1.02)	.129
Long-acting group	0.93 (0.78–1.10)	.383	0.85 (0.71–1.02)	.080
Ref: Intermediate-acting group				
Long-acting group	0.98 (0.81–1.18)	.813	0.92 (0.76–1.13)	.430

Multivariable analysis was adjusted for age; sex; body mass index; vascular access type; diabetes; HD vintage; CCI score; ultrafiltration volume; Kt/V_urea_; hemoglobin, serum albumin, serum creatinine, serum phosphorus and serum calcium levels; use of RASB, statin, clopidogrel or aspirin; presence of MI or CHF; ESA dose per week; ERI; transferrin saturation rate; and ferritin levels. Mortality was evaluated using patients with any-malignancy.

CI, confidence interval.

### Subgroup analysis and malignancy types

To determine the specific groups in which the effects of ESA types were more pronounced, we conducted subgroup analyses based on sex, age (<60 or ≥60 years, defined as the median age) and ESA dose (<5660 or ≥5660 IU/week, defined as the mean ESA dose). The hazard effect of long-acting ESAs was more prominent in subgroups with males, older individuals and those receiving high ESA doses (Table 3). However, a significant interaction was observed only in the ESA dose subgroup (*P*-value for interaction in multivariable analyses: 0.120 for sex, 0.380 for age and 0.004 for ESA dose).

**Table 3:** ESA types and HR of any malignancy according to subgroups.

	Univariable		Multivariable			Univariable		Multivariable	
	HR (95% CI)	*P*	HR (95% CI)	*P*		HR (95% CI)	*P*	HR (95% CI)	*P*
Males					Females				
Ref: Short-acting									
Intermediate-acting	1.05 (0.95–1.15)	.356	1.03 (0.93–1.14)	.580		1.01 (0.90–1.13)	.891	1.01 (0.89–1.15)	.846
Long-acting	1.31 (1.11–1.53)	.001	1.33 (1.12–1.57)	<.001		0.97 (0.78–1.22)	.805	1.02 (0.80–1.29)	.904
Ref: Intermediate-acting									
Long-acting	1.25 (1.05–1.49)	.014	1.29 (1.07–1.55)	.007		0.96 (0.76–1.23)	.768	1.00 (0.77–1.30)	.987
<60 years old					≥60 years old				
Ref: Short-acting									
Intermediate-acting	1.04 (0.93–1.17)	.488	1.05 (0.93–1.19)	.454		0.99 (0.90–1.10)	.899	1.01 (0.91–1.12)	.862
Long-acting	1.19 (0.97–1.46)	.098	1.21 (0.97–1.50)	.086		1.12 (0.95–1.33)	.185	1.20 (1.01–1.44)	.043
Ref: Intermediate-acting									
Long-acting	1.14 (0.91–1.43)	.245	1.15 (0.91–1.46)	.235		1.13 (0.94–1.36)	.201	1.19 (0.98–1.45)	.075
Low ESA dose					High ESA dose				
Ref: Short-acting									
Intermediate-acting	0.95 (0.86–1.05)	.345	0.94 (0.84–1.05)	.251		1.14 (1.02–1.27)	.018	1.15 (1.02–1.29)	.019
Long-acting	1.10 (0.95–1.28)	.300	1.09 (0.93–1.27)	.300		1.73 (1.29–2.32)	<.001	1.71 (1.26–2.32)	<.001
Ref: Intermediate-acting									
Long-acting	1.16 (0.98–1.37)	.085	1.16 (0.97–1.38)	.099		1.52 (1.12–2.06)	.007	1.49 (1.09–2.05)	.013

Multivariable analysis was adjusted for age; sex; body mass index; vascular access type; diabetes; HD vintage; CCI score; ultrafiltration volume; Kt/V_urea_; hemoglobin, serum albumin, serum creatinine, serum phosphorus and serum calcium levels; use of RASB, statin, clopidogrel or aspirin; presence of MI or CHF; ESA dose per week; ERI; transferrin saturation rate; and ferritin levels.

CI, confidence interval.

We categorized patients by ESA type and dose tertiles and then conducted both univariable and multivariable Cox regression analyses. Supplementary data, Fig. S1 illustrates the results. Although the overall association with malignancy incidence was not statistically significant, Supplementary data, Fig. S1 depicts that ESA type may have a greater influence than dose.

Supplementary data, Table S3 shows the distribution of malignancies observed across the three groups during the follow-up period. The most common sites of malignancy were kidney, lung and liver in the short-, intermediate- and long-acting groups, respectively. Huang *et al*. analyzed claims data from Taiwan, reporting that the most common cancers were genitourinary, followed by liver and colon cancers. In our study, the most common malignancies were genitourinary cancers (kidney, 14.5%; bladder, 4.5%), followed by liver and colorectal cancers, showing a similar pattern [23]. These aspects may provide a basis for the generalizability of our data to the Asian population.

### Analyses using balanced cohort

Significant differences were observed in all variables among the three groups in the original cohort. We then performed analyses using the cohort after propensity score weighting. Balance among the three groups was assessed by calculating the maximum pairwise absolute standardized mean differences (ASMDs) of the covariates before and after balancing (Supplementary data, Fig. S2). After weighting, the maximum ASMDs and baseline characteristics differences were reduced for most covariates. The numbers of patients in the short-, intermediate- and long-acting groups using the weighted cohort were 33 737, 31 346 and 23 461, respectively. Supplementary data, Table S4 shows the baseline characteristics after weighting, with attenuated differences in characteristics.

The 5-year any malignancy-free rates for the short-, intermediate- and long-acting groups were 88.4%, 88.0% and 83.4%, respectively (Supplementary data, Fig. S3: *P* = .012 for trends; *P* = .297 for short- vs intermediate-acting group; *P* = .004 for short- vs long-acting group; *P* = .011 for intermediate- vs long-acting group). The 5-year patient survival rates for the short-, intermediate- and long-acting groups were 40.2%, 43.7% and 44.2%, respectively (*P* = .274 for trends; *P* = .241 for short- vs intermediate-acting group; *P* = .253 for short- vs long-acting group; *P* = .291 for intermediate- vs long-acting group). Univariable and multivariable analyses of any malignancy indicated that the HR in the long-acting group was higher than that in the short- or intermediate-acting groups (Supplementary data, Table S5).

## DISCUSSION

In a study involving 33 960 HD patients prescribed ESA, the use of long-acting ESA was associated with increased any malignancy incidence, although no difference in mortality rates was observed. The use of long-acting ESA increased the risk of any malignancy by 21% in the short-acting ESA group and 17% in the intermediate-acting ESA group. Subgroup analysis revealed that, among patients administered high doses of ESA, the incidence of any malignancy was significantly higher in those using long-acting ESA than in those using other ESA formulations. Further analyses using a balanced cohort, which attenuated baseline characteristics differences, confirmed that the high malignancy incidence in the long-acting group was maintained.

ESAs induce red blood cell production by acting on erythropoietin receptors. Beyond this beneficial effect, previous studies have demonstrated that ESAs promote angiogenesis and tumor growth, raising significant concerns about their use in patients at risk for cancer or those with pre-existing cancer [24–26]. However, the relationship between ESA use and malignancy risk remains inconsistent in the literature. Two meta-analyses indicate no association between ESA use and mortality in patients with solid organ cancers, while studies in dialysis patients suggest that high-dose ESA use increases cancer incidence [27–29]. ESAs are classified based on half-life: short-acting, intermediate-acting and long-acting agents. Although some studies comparing continuous erythropoietin receptor activators and epoetin-alfa in dialysis patients have included malignancy incidence as a safety concern, the number of malignancies in these studies is limited to one to two cases per group, making meaningful comparisons challenging [27].

Our study aims to determine whether malignancy incidence differs based on half-life of ESAs. In clinical practice, short-acting ESAs (epoetin alfa, beta, or delta) are typically administered two to three times per week, intermediate-acting ESAs (darbepoetin-alfa) 2–4 times per months, and long-acting ESAs (methoxy polyethylene glycol-epoetin beta) once or twice per month. Patients were classified into three groups, and the data were analyzed. Our findings revealed that the long-acting ESA group had a higher incidence of any malignancy than those of the other groups. During the follow-up period, malignancy incidence was 16.0%, 16.1% and 21.6% in the short-, intermediate- and long-acting ESA groups, respectively.

Functional erythropoietin receptors have been identified on erythroid progenitor cells and in various cancer tissues, including those of the bladder, breast, cervix, gastrointestinal tract, head and neck, and kidney [30, 31]. Although the stimulatory effects of erythropoietin on non-hematopoietic tissues remain a subject of debate, the expression of functional receptors provides a potential biological basis for concern. Short-acting ESAs generally exhibit higher receptor affinity but shorter receptor engagement, while long-acting ESAs are characterized by prolonged receptor activation despite having lower affinity. Prolonged erythropoietin receptor activation may theoretically sustain signaling pathways involved in angiogenesis, cell survival and proliferation**—**processes implicated in tumorigenesis. However, limited data exist on the association between ESA types or duration of action and cancer risk, and the biological mechanisms linking the two factors remain hypothetical. Therefore, our findings should be interpreted as exploratory and hypothesis-generating, rather than as definitive evidence of causality. Further mechanistic studies are needed to determine whether differences in ESA pharmacodynamics contribute to cancer risk in patients undergoing maintenance HD.

In the high-dose ESA subgroup, the long-acting group exhibited a significantly higher cancer risk than that of the short- or intermediate-acting group. Additionally, a statistically significant interaction was observed between ESA dose and malignancy risk. A previous study revealed a relationship between high-dose ESA and an increased malignancy risk [27]. Our findings confirm this association, revealing that the high-dose subgroup had a greater malignancy risk, which was further amplified in patients using long-acting ESAs. Therefore, long-acting ESAs may be avoided in patients requiring high-dose, while low doses can be used irrespectively of the ESA type. However, further research is needed to substantiate this hypothesis. While not statistically significant, a trend toward a higher malignancy risk was observed in male and elderly patients. This may reflect the inherently higher baseline malignancy risk in these subgroups than in females or younger patients, which could lead to greater statistical significance in these populations.

Unlike its association with malignancy risk, ESA type did not significantly influence survival rates in patients who developed malignancy. Here, ESA groups were defined based on ESA use during the 6-month HD quality assessment period. Changes in ESA type, use or dose after malignancy diagnosis may have influenced the results, as ESA use is likely to have been discontinued following the diagnosis of malignancy. Furthermore, factors such as cancer stage, treatment modalities, and patient performance status likely had a stronger influence on survival outcomes than ESA type.

Although our study focused on patients undergoing maintenance HD for at least 6 months during the program period, those who underwent HD via a catheter were excluded. HD catheter use may indicate incident patients who are new to HD or those with the potential for renal recovery, which could lead to discontinuation of dialysis after the program period. Moreover, catheter use is associated with increased inflammation, which could influence ESA-type selection and dosing, thereby introducing potential confounding [32]. Therefore, we excluded these patients to minimize potential bias. Additionally, since our study utilized a population-based database from South Korea, some patients who enrolled in the fourth HD quality assessment program were also included in the fifth program. In cases where patients were enrolled in both programs, we used only data from the fourth program enrollment. Consequently, those initially included in the fourth program were excluded from the fifth program cohort analysis.

In our study, fewer patients used long-acting ESAs compared with those using short- or intermediate-acting ESAs. This may be attributed to the unique reimbursement system and strict hemoglobin target requirement under the HD quality assessment program in South Korea. In South Korea, the reimbursement for HD is bundled, with a fixed payment of approximately 136 000–146 210 KRW per HD session, covering dialysis procedures, prescribed medications, laboratory studies, treatment fees and material costs [33]. Under this system, minimizing medication costs per session is critical for optimizing revenue. For instance, short-acting ESAs administered at a dose of 6000 IU per week (2000 IU per HD session, three times per week) cost approximately 9000 KRW per administration. In contrast, darbepoetin-alfa at an equivalent dose (30 μg) costs approximately 31 000 KRW per administration, while long-acting ESAs at a corresponding dose (100 μg) cost approximately 114 000 KRW per administration. Consequently, most HD centers prefer short- or intermediate-acting ESAs over long-acting ESAs. In addition, the HD Quality Assessment Program in South Korea mandates maintaining hemoglobin levels above 10 g/dL, with performance incentives linked to this target. Moreover, ESA prescriptions are not reimbursed by insurance if the hemoglobin level of a patient exceeds 11 g/dL, requiring patients to cover 100% of the cost out-of-pocket. Since hemoglobin testing is relatively inexpensive (approximately 1000 KRW per test), clinicians frequently monitor hemoglobin levels to maintain them slightly above 10 g/dL, avoiding excessive elevations that would necessitate ESA discontinuation. When hemoglobin levels drop below the target range, prompt intervention is necessary to rapidly increase the hemoglobin concentration. These unique clinical and economic factors likely contributed to the observed imbalance in ESA utilization. Furthermore, long-acting ESAs more commonly prescribed in settings such as tertiary hospitals, where there cost sensitivity is lower to medication costs than in private HD centers. We cannot exclude the possibility that this imbalance may have influenced our ability to assess the independent effect of ESA type on malignancy risk.

A clustering of malignancy events was observed for approximately 12 months in the Kaplan–Meier curve for the long-acting group. However, given the relatively small sample size in this group, this observation may be influenced by a few coincidental cases rather than a true biological phenomenon. No known biological mechanism suggests that long-acting ESA use specifically increases malignancy risk at approximately 12 months. Therefore, this observation is likely attributable to random variation rather than a true time-dependent effect.

Our study findings should be interpreted with caution in terms of generalizability. The association between ESA use and patient outcomes may vary depending on usage patterns, ethnicity and national healthcare systems. Previous studies report inconsistent findings regarding the relationship between ESA type and mortality across different countries, likely due to variations in clinical practice, patient demographics and healthcare policies. For example, an analysis of the Japanese Society for Dialysis Therapy Renal Data Registry database demonstrated higher mortality rates in patients undergoing long-acting ESAs than those of short-acting ESAs [34]. In contrast, analyses of the United States Renal Data System database and the multinational Dialysis Outcomes and Practice Pattern Study data showed no significant association between ESA type and mortality [35, 36]. Our study, based on a large nationwide cohort within the South Korean healthcare system, provides valuable insights into real-world outcomes in this context. However, given the unique features of the South Korean healthcare system and clinical practices, these findings may not be directly applicable to other populations or healthcare environments. Therefore, caution is warranted when applying these results to different ethnicities and nations. Further multinational and mechanistic studies are needed to validate and deepen our understanding of the potential effect of ESA type across diverse healthcare settings.

Our study has some limitations. First, it was retrospective, and the baseline characteristics and group sizes differed significantly among the three groups, likely owing to selection bias. However, we mitigated these differences through multivariable and subgroup analyses, as well as analyses using a balanced cohort. Second, specific data related to malignancy, such as stage or treatment, were unavailable. Malignancy stage information is crucial for malignancy-associated studies. However, our study was based on data collected for assessing the adequacy of HD facilities and claims data from these patients. Therefore, detailed information beyond the occurrence of malignancy during the follow-up period was unavailable. Despite this limitation, the reliability of malignancy diagnoses in this dataset is considered high. In South Korea, ICD-10 codes for malignancy require additional details such as malignancy type and diagnostic methods. Furthermore, patients with these codes benefit significantly, including reduced out-of-pocket medical expenses for over 5 years through the special exemption policy and eligibility for disability registration. Consequently, the input of ICD-10 codes for malignancy is strictly managed.

In conclusion, our population-based cohort study reveals an association between long-acting ESAs use and the incidence of any malignancy, with a particularly strong influence on high-dose users. This suggests that avoiding long-acting ESAs may be advisable for patients at high risk of malignancy. However, owing to the various limitations of this study, further research is needed to confirm this association more definitively.

## Supplementary Material

sfaf148_Supplemental_File

## Data Availability

Raw data were generated at the Health Insurance Review and Assessment Service. The database can be requested from the Health Insurance Review and Assessment Service by sending a study proposal, including the purpose of the study, study design and duration of analysis at the website: https://www.hira.or.kr. The authors cannot distribute the data without permission.
