# Supplementary material for: Effect of erythropoiesis-stimulating agent types on malignancy in hemodialysis patients
Source: Clin Kidney J. 2025 Jun 16;18(6):sfaf148. doi: 10.1093/ckj/sfaf148 (PMC12199780; doi:10.1093/ckj/sfaf148)
Supplement: sfaf148_Supplemental_File [file sfaf148_supplemental_file.docx]

**Table S1.** Medication types and Health Insurance Review and Assessment Service codes

**Table S2.** Baseline characteristics according to different HD quality assessment programs

**Table S3.** Distribution of malignancies observed across groups

**Table S4.** Baseline characteristics using a balanced cohort

**Table S5.** ESA types and HR of any malignancy using a balanced cohort

**Figure S1.** Hazard ratios for any malignancy based on ESA type and dose

**Figure S2.** Balance tests.

**Figure S3.** Kaplan–Meier curves by type of erythropoiesis stimulating agents using a balanced cohort

**Table S1. Medication types and Health Insurance Review and Assessment Service codes**

| **Medications** | **Codes** |
| --- | --- |
| **Alacepril** | 104201ATB, 104202ATB |
| **Benazepril** | 114701ATB |
| **Captopril** | 122901ATB, 122902ATB, 122903ATB |
| **Cilazapril** | 133001ATB, 133002ATB, 133003ATB |
| **Enalapril** | 151601ATB, 151603ATB |
| **Fosinopril** | 163501ATB, 163502ATB |
| **Imidapril** | 173401ATB, 173402ATB |
| **Moexipril** | 196801ATB, 196802ATB |
| **Lisinopril** | 184501ATB |
| **Perindopril** | 211301ATB, 211302ATB, 501601ATB, 501602ATB |
| **Quinapril** | 221901ATB, |
| **Ramipril** | 222401ATB, 222402ATB, 222404ATB |
| **Zofenopril** | 510401ATB, 510402ATB, 510403ATB |
| **Temocapril** | 235002ATB |
| **Delapril** | 140901ATB, 140902ATB |
| **Captopril + Hydrochlorothiazide** | 262200ATB, 262300ATB |
| **Enalapril + Hydrochlorothiazide** | 440300ATB, 453700ATB, 453600ATB |
| **Ramipril + Felodipine** | 447100ATB, 447200ATB |
| **Ramipril + Hydrochlorothiazide** | 448600ATB, 448700ATB |
| **Perindopril + indapamide** | 556200ATB |
| **Lisinopril + Hydrochlorothiazide** | 499200ATB, 499300ATB |
| **Moexipril + Hydrochlorothiazide** | 440800ATB, 497900ATB |
| **Enalapril + nitrendipine** | 466000ATB |
| **Candesartan** | 122601ATB, 122602ATB, 122603ATB |
| **Irbesartan** | 177301ATB, 177303ATB |
| **Losartan** | 185701ATB, 185702ATB |
| **Valsartan** | 247101ATB, 247102ATB, 247103ATB, 247104ATB |
| **Fimasartan** | 515201ATB, 515202ATB, 515203ATB |
| **Azilsartan** | 662401ATB, 662402ATB, 662403ATB |
| **Telmisartan** | 378801ATB, 378802ATB |
| **Eprosartan** | 429201ATB |
| **Olmesartan** | 468501ATB, 468502ATB, 468503ATB, 520901ATB, 520902ATB |
| **Valsartan + Amlodipine** | 492800ATB, 492900ATB, 495800ATB, 522600ABTB, 522700ABTB, 522800ABTB, 522900ABTB, 523000ATB, 523100ATB, 523200ATB, 523300ATB, 523400ATB |
| **Valsartan + Lercanidipne** | 522200ATB. 522300ATB. 522400ATB |
| **Valsartan + Pitavastatin** | 634900ATB, 635000ATB, 635100ATB, 635200ATB |
| **Valsartan + Sacubitril** | 651401ATB, 651402ATB, 651403ATB |
| **Valsartan + Rosuvastatin** | 629700ATB, 629800ATB, 525000ATB, 525100ATB, 525200ATB, 525300ATB, |
| **Valsartan + Hydrochlorothiazide** | 356400ATB, 442600ATB |
| **Olmesaetan + Amlodipine** | 500500ATB, 500600ATB, 547500ATB. 547600ATB, 547700ATB, 547800ATB, 547900ATB, 548000ATB, 582200ATB, 582400ATB, 629400ATB, 629500ATB, 629600ATB, 631300ATB, 632800ATB, 632900ATB, 633000ATB |
| **Olmesartan + Hydrochlorothiazide** | 513600ATB |
| **Olmesartan + Hydrochlorothiazide + Amlodipine** | 519700ATB, 519800ATB, 519900ATB, 520000ATB, 520100ATB |
| **Olmesartan + Rosuvastatin** | 653200ATB, 644100ATB, 644200ATB, 526300ATB, 526400ATB, 526500ATB, 526900ATB |
| **Telmisartan + Hydrochlorothiazide** | 502600ATB, 443200ATB, 443300ATB |
| **Telmisartan + Rosuvastatin** | 629900ATB, 630000ATB, 630100ATB, 630200ATB, 631600ATB, 631700ATB |
| **Telmisartan + Amlodipine** | 511500ATB, 511600ATB, 511700ATB, 521200ATB, 521300ATB, 521400ATB, 623100ATB, 644800ATB |
| **Telmisartan+ Hydrochlorothiazide + Amlodipine** | 663500ATB, 663600ATB, 663700ATB, 663800ATB |
| **Telmisartan + Rosuvastatin + Amlodipine** | 671700ATB, 671600ATB, 671500ATB, 671400ATB, 671300ATB, 671200ATB, |
| **Losartan + Hydrochlorothiazide** | 262500ATB, 378900ATB, 486900ATB |
| **Losartan + Amlodipine** | 502700ATB, 503000ATB, 513900ATB, 637400ATB, 637500ATB, 637600ATB |
| **Losartan+Rosuvastatin+ Amlodipine** | 663900ATB, 664000ATB, 664100ATB, 664200ATB, 664300ATB, 664400ATB, |
| **Losartan+Hydrochlorothiazide+ Amlodipine** | 662800ATB, 662900ATB, 663000ATB |
| **Fimasartan + Hydrochlorothiazide** | 522000ATB, 526800ATB |
| **Fimasartan + Amlodipine** | 651900ATB, 652000ATB, 652100ATB, 652700ATB, 651900ATB |
| **Fimasartan + Rosuvastatin** | 654600ATB, 654700ATB, 654800ATB, 654900ATB, 655000ATB |
| **Candesartan + Hydrochlorothiazide** | 423700ATB |
| **Candesartan + Amlodipine** | 652900ATB, 653000ATB, 653100ATB, 652900ATB, 652900ATB |
| **Candesartan + Rosuvastatin** | 673700ATB, 661800ATB, 661900ATB, 662000ATB, 662100ATB |
| **Irbesartan + Hydrochlorothiazide** | 385700ATB, 385800ATB |
| **Irbesartan + Atorvastatin** | 527000ATB, 527100ATB, 524000ATB, 524100ATB |
| **Azilsartan + Chlorthalidone** | 673500ATB, 673600ATB |
| **Eprosartan + Hydrochlorothiazide** | 460500ATB |
| **Amlodipine + Atorvastatin** | 614500ATB, 472300ATB, 472400ATB, 472500ATB, 518900ATB |
| **Amlodipine + Rosuvastatin** | 673900ATB, 674000ATB, 674100ATB |
| **Atorvastatin + Ezetimibe** | 633800ATB, 633900ATB, 634800ATB |
| **Pitavastatin + Fenofibrate** | 679300ACH |
| **Rosuvastatin + Ezetimibe** | 640700ATB, 640800ATB, 640900ATB |
| **Metformin + Atorvastatin** | 671800ATR, 673800ATR, 671900ATR, 672000ATR, 672100ATR |
| **Metformin + Rosuvastatin** | 672500ATR, 672600ATR, 672700ATR, 672800ATR, 672900ATR, 673000ATR, 683300ATR, 683400ATR |
| **Gemigliptin + Rosuvastatin** | 664600ATB, 664700ATB, 664800ATB |
| **Aspirin** | 110701ATB, 110702ATB, 110801ATB, 110802ATB, 111001ACE, 111001ATB, 111001ATE, 111002ATE, 111003ACE, 111003ATE |
| **Clopidogrel** | 133201ACR, 133201ATB, 133201ATR, 133202ATB, 133203ATR, 506100ATB |
| **Cilostazol** | 136901ATB, 492501ATB, 495201ATB, 498801ATB, 501501ATB |
| **Ticlopidine** | 498900ATB, 239201ATB, 239202ATB |
| **Aspirin + Bethocarbamol** | 256800ATB |
| **Aspirin + Clopidogrel** | 517900ACH, 517900ACE, 517900ATE, 667500ACE |
| **Aspirin + Dipyridamole** | 489700ACR |
| **Atorvastatin** | 111502ATB, 502202ATB, 633900ATB, 472400ATB, 518900ATB, 524100ATB, 527000ATB, 672000ATR, 672100ATR, 111503ATB, 502203ATB, 634800ATB, 472500ATB, 111504ATB, 502204ATB |
| **Fluvastatin** | 162401ACH, 162402ACH, 162403ATR |
| **Lovastatin** | 185801ATB |
| **Pitavastatin** | 470901ATB, 470902ATB, 470903ATB |
| **Pravastatin** | 216601ATB, 216602ATB, 216603ATB, 216604ATB |
| **Rosuvastatin** | 454001ATB, 454002ATD, 454002ATB, 454003ATB, 454003ATD, 454005ATB |
| **Simvastatin** | 227801ATB, 227802ATB, 227803ATB, 227805ATB, 227806ATB |
| **Darbepoetin α** | 455738BIJ, 455739BIJ, 455735BIJ, 455736BIJ, 455737BIJ, 455702BIJ, 455703BIJ, 455704BIJ, 455707BIJ, 455708BIJ |
| **Erythropoietin** | 500334BIJ, 500337BIJ, 500340BIJ, 500341BIJ, 500342BIJ, 500343BIJ, 500330BIJ, 500332BIJ, 500331BIJ, 500333BIJ, 500335BIJ, 500338BIJ, 500336BIJ, 500339BIJ, 154305BIJ, 154308BIJ, 154303BIJ, 154306BIJ, 154309BIJ, 154301BIJ, 154302BIJ, 500301BIJ, 500302BIJ, 500303BIJ, 500304BIJ, 500305BIJ, 500306BIJ, 500307BIJ, 500308BIJ |
| **Methoxyl polyethylene glycol-epoetin β** | 504302BIJ, 504303BIJ, 504301BIJ, 504305BIJ, 504304BIJ, 504306BIJ, 504307BIJ, 504308BIJ, 504309BIJ, 504311BIJ, 504310BIJ |

**Table S2. Baseline characteristics according to different HD quality assessment programs**

|  | **4^th^ Program** | | | | **5^th^ Program** | | | | |  |
| --- | --- | --- | --- | --- | --- | --- | --- | --- | --- | --- |
|  | **Short-acting**  **(n = 11,853)** | **Intermediate-acting**  **(n = 2,757)** | **Long-acting**  **(n = 603)** | ***P*-value** | | **Short-acting**  **(n = 14,153)** | **Intermediate-acting**  **(n = 3,691)** | **Long-acting**  **(n = 903)** | ***P*-value** | |
| Age (years) | 60.1 ± 12.8 | 61.4 ± 12.8^a^ | 60.7 ± 12.9 | <0.001 | | 60.2 ± 12.9 | 60.5 ± 13.0 | 62.0 ± 12.3^ab^ | <0.001 | |
| Sex (male, %) | 6,736 (56.8%) | 1,477 (53.6%) | 355 (58.9%) | 0.004 | | 8,370 (59.1%) | 2,101 (56.9%) | 509 (56.4%) | 0.020 | |
| Hemodialysis vintage (months) | 67 ± 60 | 60 ± 54^a^ | 62 ± 61 | <0.001 | | 60 ± 66 | 53 ± 59^a^ | 50 ± 58^a^ | <0.001 | |
| Body mass index (kg/m^2^) | 22.2 ± 3.2 | 22.3 ± 3.3 | 22.5 ± 3.2^a^ | 0.039 | | 22.4 ± 3.4 | 22.6 ± 3.6^a^ | 22.8 ± 3.3^a^ | <0.001 | |
| Diabetes (%) | 5,005 (42.2%) | 1,284 (46.6%) | 261 (43.3%) | <0.001 | | 6,394 (45.2%) | 1,707 (46.2%) | 438 (48.5%) | 0.096 | |
| CCI score | 5.8 ± 2.3 | 5.9 ± 2.3 | 6.1 ± 2.6^ab^ | <0.001 | | 7.4 ± 2.6 | 7.2 ± 2.5^a^ | 7.5 ± 2.5^b^ | 0.014 | |
| Arteriovenous fistula (%) | 10,206 (86.1%) | 2,333 (84.6%) | 529 (87.7%) | 0.055 | | 12,032 (85.0%) | 3,120 (84.5%) | 754 (83.5%) | 0.393 | |
| Kt/V_urea_ | 1.50 ± 0.26 | 1.55 ± 0.29^a^ | 1.53 ± 0.27^a^ | <0.001 | | 1.53 ± 0.27 | 1.56 ± 0.29^a^ | 1.58 ± 0.27^a^ | <0.001 | |
| Ultrafiltration volume (L/session) | 2.31 ± 1.01 | 2.24 ± 1.07^a^ | 2.23 ± 1.08 | 0.002 | | 2.31 ± 0.91 | 2.27 ± 0.94 | 2.21 ± 0.95^a^ | 0.002 | |
| Hemoglobin (g/dL) | 10.6 ± 0.7 | 10.6 ± 0.7^a^ | 10.7 ± 0.8^a^ | <0.001 | | 10.6 ± 0.7 | 10.7 ± 0.7^a^ | 10.7 ± 0.8^a^ | <0.001 | |
| Serum albumin (g/dL) | 4.00 ± 0.34 | 3.93 ± 0.33^a^ | 3.96 ± 0.33^a^ | <0.001 | | 3.99 ± 0.34 | 3.93 ± 0.34^a^ | 3.94 ± 0.31^a^ | <0.001 | |
| Serum phosphorus (mg/dL) | 5.05 ± 1.39 | 4.89 ± 1.30^a^ | 5.05 ± 1.42^b^ | <0.001 | | 4.94 ± 1.29 | 4.77 ± 1.27^a^ | 4.90 ± 1.36^b^ | <0.001 | |
| Serum calcium (mg/dL) | 8.94 ± 0.90 | 8.88 ± 0.77^a^ | 8.95 ± 0.80 | 0.002 | | 8.94 ± 0.81 | 8.87 ± 0.68^a^ | 8.80 ± 0.81^ab^ | <0.001 | |
| Serum creatinine (mg/dL) | 9.6 ± 2.7 | 9.5 ± 2.7 | 9.4 ± 2.6^a^ | 0.012 | | 9.5 ± 2.7 | 9.4 ± 2.7^a^ | 8.9 ± 2.7^ab^ | <0.001 | |
| Use of RASB (%) | 8,164 (68.9%) | 2,001 (72.6%) | 432 (71.6%) | <0.001 | | 9,808 (69.3%) | 2,656 (72.0%) | 631 (69.9%) | 0.007 | |
| Use of aspirin (%) | 1,464 (12.4%) | 268 (9.7%) | 63 (10.4%) | <0.001 | | 1,986 (14.0%) | 429 (11.6%) | 145 (16.1%) | <0.001 | |
| Use of clopidogrel (%) | 900 (7.6%) | 149 (5.4%) | 38 (6.3%) | <0.001 | | 1,231 (8.7%) | 223 (6.0%) | 74 (8.2%) | <0.001 | |
| Use of statins (%) | 4,359 (36.8%) | 1,225 (44.4%) | 267 (44.3%) | <0.001 | | 6,172 (43.6%) | 1,726 (46.8%) | 404 (44.7%) | 0.003 | |
| MI or CHF | 3,579 (30.2%) | 858 (31.1%) | 181 (30.0%) | 0.625 | | 6,574 (46.4%) | 1,802 (48.8%) | 423 (46.8%) | 0.037 | |
| Transferrin saturation (%) | 32.8 ± 14.3 | 33.5 ± 15.0 | 36.5 ± 14.8^ab^ | <0.001 | | 32.6 ± 13.8 | 32.8 ± 14.9 | 35.7 ± 14.0^ab^ | <0.001 | |
| Ferritin | 263 ± 256 | 299 ± 291^a^ | 269 ± 269^b^ | <0.001 | | 260 ± 263 | 279 ± 285^a^ | 269 ± 223 | <0.001 | |
| ERI [(IU/week)/kg/(g/dL)] | 10.6 ± 6.4 | 9.6 ± 6.8^a^ | 5.9 ± 4.2^ab^ | <0.001 | | 10.4 ± 6.5 | 9.7 ± 7.3^a^ | 6.2 ± 3.7^ab^ | <0.001 | |

Data are expressed as means ± standard deviation for continuous variables and as numbers (percentages) for categorical variables. *P*-values were tested using one-way analysis of variance, followed by the Tukey post hoc test. Pearson’s χ^2^ test was performed for categorical variables. ^a^*P* < 0.05 vs. Short acting, ^b^*P* < 0.05 vs. Intermediate acting.

**Abbreviations:** CCI, Charlson comorbidity index; CHF, congestive heart failure; ERI, erythropoietin resistance index; MI, myocardial infarction; RASB, renin–angiotensin system blocker.

**Table S3. Distribution of malignancies observed across groups**

|  | **Total** | **Short-acting** | **Intermediate-acting** | **Long-acting** |
| --- | --- | --- | --- | --- |
| **Total** | 5529 | 4169 | 1035 | 325 |
| Thyroid | 425 (7.7%) | 314 (7.5%) | 93 (9.0%) | 18 (5.5%) |
| Lung | 698 (12.6%) | 505 (12.1%) | 157 (15.2%) | 36 (11.1%) |
| Colorectum | 737 (13.3%) | 563 (13.5%) | 132 (12.8%) | 42 (12.9%) |
| Stomach | 549 (9.9%) | 429 (10.3%) | 94 (9.1%) | 26 (8.0%) |
| Breast | 266 (4.8%) | 186 (4.5%) | 63 (6.1%) | 17 (5.2%) |
| Prostate | 399 (7.2%) | 303 (7.3%) | 73 (7.1%) | 23 (7.1%) |
| Liver | 749 (13.5%) | 564 (13.5%) | 118 (11.4%) | 67 (20.6%) |
| Pancreas | 329 (6.0%) | 253 (6.1%) | 64 (6.2%) | 12 (3.7%) |
| Gall bladder and bile duct | 165 (3.0%) | 123 (3.0%) | 34 (3.3%) | 8 (2.5%) |
| Kidney | 804 (14.5%) | 608 (14.6%) | 141 (13.6%) | 55 (16.9%) |
| Uterus or cervix | 159 (2.9%) | 122 (2.9%) | 28 (2.7%) | 9 (2.8%) |
| Bladder | 249 (4.5%) | 199 (4.8%) | 38 (3.7%) | 12 (3.7%) |

**Table S4. Baseline characteristics using balanced cohort**

|  | **Short-acting**  **(n = 33,737)** | **Intermediate-acting**  **(n = 31,346)** | **Long-acting**  **(n = 23,461)** | ***P*-value** |
| --- | --- | --- | --- | --- |
| Age (years) | 60.3 ± 0.1 | 60.5 ± 0.2 | 60.7 ± 0.6 | 0.299 |
| Sex (male, %) | 19,506 (57.8%) | 17,868 (57.0%) | 13,837 (59.0%) | 0.461 |
| Hemodialysis vintage (months) | 61 ± 0 | 59 ± 1 | 57 ± 3 | 0.022 |
| Body mass index (kg/m^2^) | 22.3 ± 0.0 | 22.4 ± 0.0 | 22.5 ± 0.1 | 0.151 |
| Diabetes (%) | 14,954 (44.3%) | 14255 (45.5%) | 10540 (44.9%) | 0.665 |
| CCI score | 6.6 ± 0.0 | 6.6 ± 0.0 | 6.9 ± 0.1 | 0.535 |
| Arteriovenous fistula (%) | 28,813 (85.4%) | 26,590 (84.8%) | 19,591 (83.5%) | 0.329 |
| Kt/V_urea_ | 1.53 ± 0.00 | 1.53 ± 0.00 | 1.52 ± 0.01 | 0.069 |
| Ultrafiltration volume (L/session) | 2.29 ± 0.01 | 2.28 ± 0.01 | 2.30 ± 0.04 | 0.430 |
| Hemoglobin (g/dL) | 10.6 ± 0.0 | 10.6 ± 0.0 | 10.6 ± 0.0 | 0.631 |
| Serum albumin (g/dL) | 3.99 ± 0.00 | 3.97 ± 0.00 | 3.93 ± 0.02 | <0.001 |
| Serum phosphorus (mg/dL) | 4.96 ± 0.01 | 4.91 ± 0.02 | 4.94 ± 0.06 | 0.006 |
| Serum calcium (mg/dL) | 8.93 ± 0.01 | 8.91 ± 0.01 | 8.84 ± 0.03 | 0.087 |
| Serum creatinine (mg/dL) | 9.5 ± 0.0 | 9.6 ± 0.0 | 9.3 ± 0.1 | 0.291 |
| Use of RASB (%) | 23,435 (69.5%) | 22,349 (71.3%) | 16,631 (70.9%) | 0.393 |
| Use of aspirin (%) | 4,389 (13.0%) | 3,678 (11.7%) | 2,831 (12.1%) | 0.387 |
| Use of clopidogrel (%) | 2,653 (7.9%) | 2,108 (6.7%) | 1,458 (6.2%) | 0.130 |
| Use of statins (%) | 13,973 (41.4%) | 13,519 (43.1%) | 9,996 (42.6%) | 0.474 |
| MI or CHF | 13,242 (39.3%) | 12,826 (40.9%) | 9,211 (39.3%) | 0.440 |
| Transferrin saturation (%) | 32.9 ± 0.1 | 33.0 ± 0.2 | 33.3 ± 0.6 | 0.487 |
| Ferritin | 266 ± 2 | 274 ± 3 | 269 ± 12 | 0.045 |
| ERI [(IU/week)/kg/(g/dL)] | 10.1 ± 0.0 | 10.1 ± 0.1 | 9.1 ± 0.3 | 0.765 |

Data are expressed as mean ± standard error for continuous variables and as numbers (percentages) for categorical variables. *P*–values are tested using general linear model using a complex survey design, including sample weights.

**Abbreviations:** CCI, Charlson comorbidity index; CHF, congestive heart failure; ERI, erythropoietin resistance index; MI, myocardial infarction; RASB, renin–angiotensin system blocker.

**Table S5. ESA types and HR of any malignancy using balanced cohort**

|  | **Univariable** | | **Multivariable** | |  |
| --- | --- | --- | --- | --- | --- |
|  | **HR (95% CI)** | ***P*-value** | **HR (95% CI)** | ***P*-value** | |
| Ref: Short-acting group |  |  |  |  | |
| Intermediate-acting group | 1.04 (0.99–1.09) | 0.052 | 1.06 (1.01–1.11) | 0.013 | |
| Long-acting group | 1.46 (1.40–1.52) | <0.001 | 1.59 (1.52–1.66) | <0.001 | |
| Ref: Intermediate-acting group |  |  |  |  | |
| Long-acting group | 1.40 (1.35–1.46) | <0.001 | 1.50 (1.43–1.57) | <0.001 | |

Multivariate analysis was adjusted for age; sex; body mass index; vascular access type; diabetes; hemodialysis vintage; Charlson Comorbidity Index score; ultrafiltration volume; Kt/V_urea_; hemoglobin, serum albumin, serum creatinine, serum phosphorus, and serum calcium levels; use of renin–angiotensin system blockers, statin, clopidogrel, or aspirin; presence of myocardial infarction or congestive heart failure; ESA dose per week; erythropoietin resistance index; transferrin saturation rate; and ferritin levels.

**Abbreviations**: CI, confidence interval; ESA, erythropoiesis stimulating agent; HR, hazard ratio.


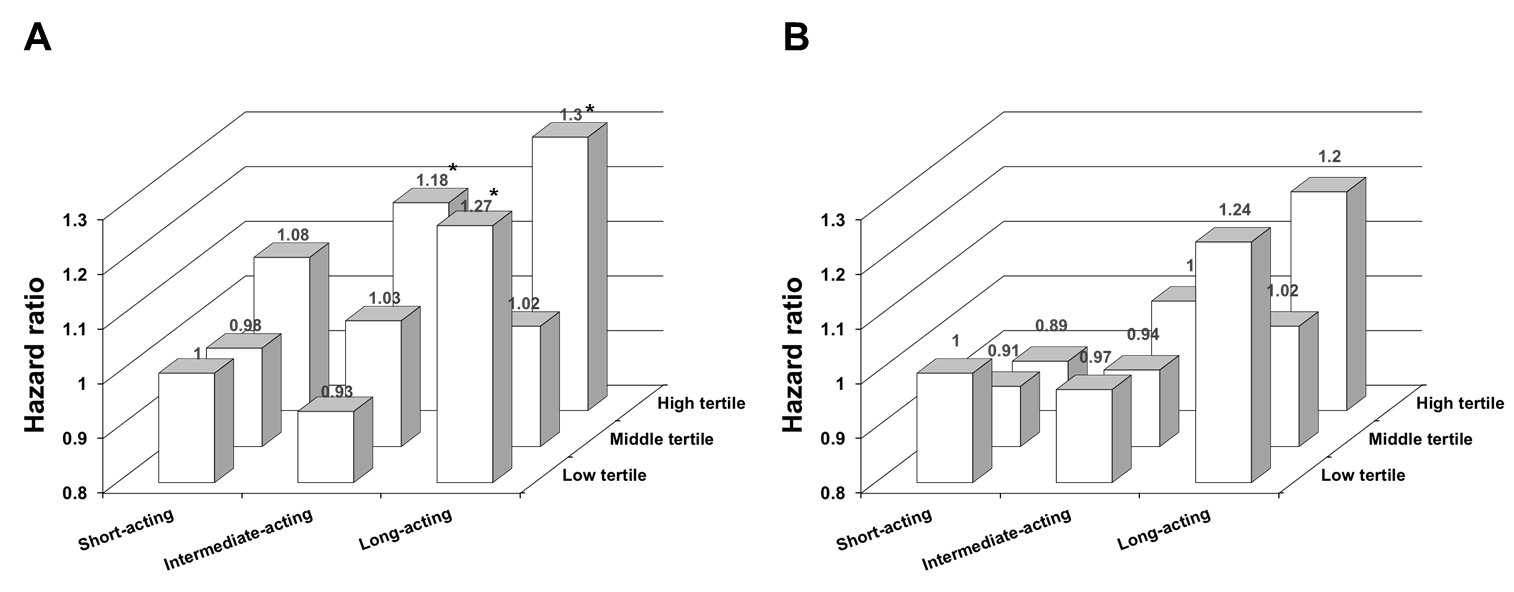


**Figure S1. Hazard ratios for any malignancy based on ESA type and dose.**

The reference group is the low tertile of short-acting ESA. (A) Univariable and (B) multivariable Cox regression analyses were performed. The multivariable model was adjusted for the following covariates: age, sex, body mass index, vascular access type, diabetes, hemodialysis vintage, Charlson Comorbidity Index score, ultrafiltration volume, Kt/V_urea_, hemoglobin, serum albumin, serum creatinine, serum phosphorus, and serum calcium levels; use of renin–angiotensin system blockers, statins, clopidogrel, or aspirin; presence of myocardial infarction or congestive heart failure; ESA dose per week, erythropoietin resistance index, transferrin saturation rate, and ferritin levels. **P* < 0.05.

**
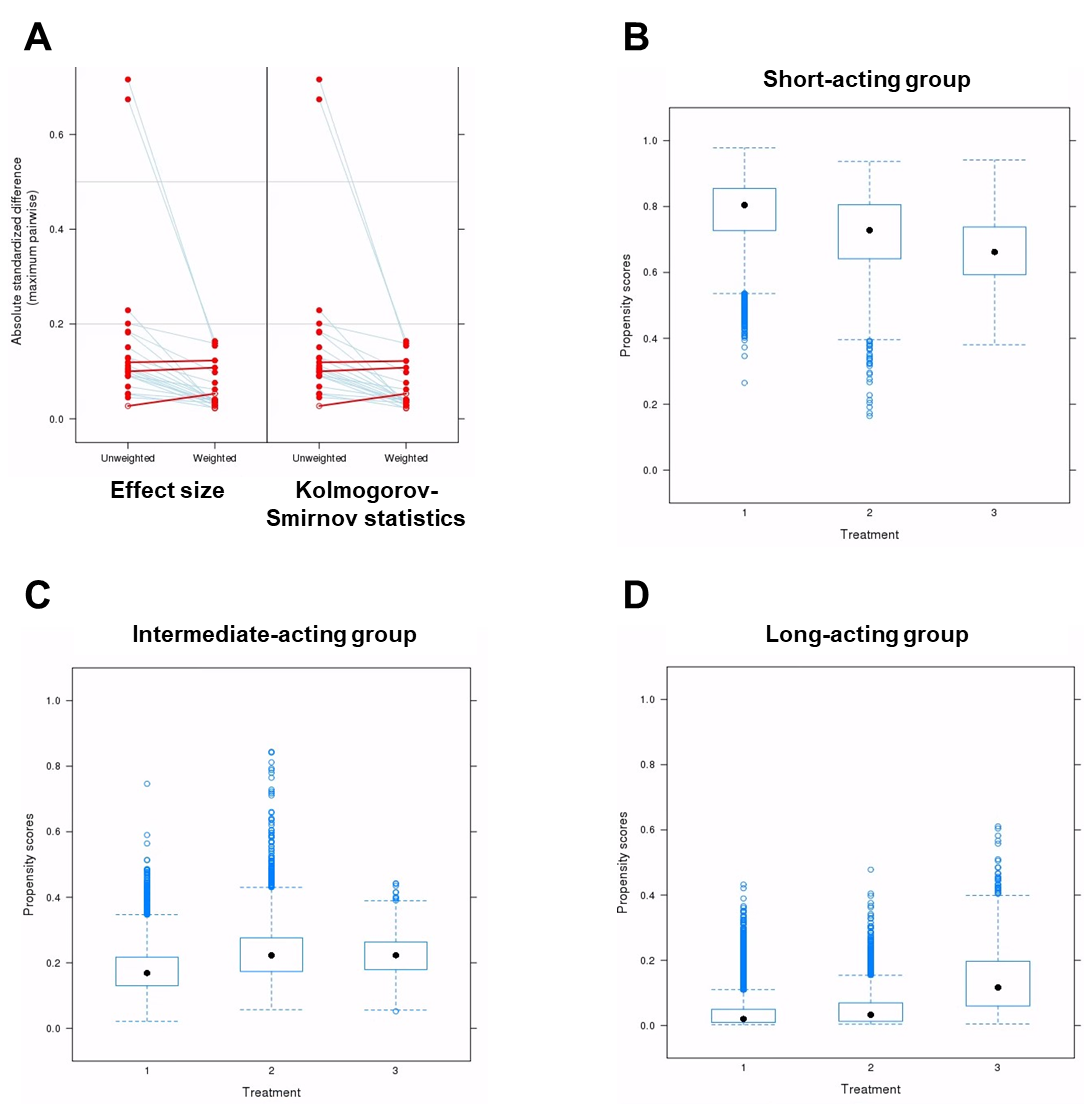
**

**Figure S2. Balance tests.** (A) Absolute standardized difference plots for estimating propensity score to generate weights. (B-D) Boxplot illustrating the spread of propensity scores by treatment group for each group (i.e. Short-, Intermediate-, or Long-acting groups). The filled black circles indicate the median propensity score in each treatment group. As the plot demonstrates, there was substantial overlap in the total spread of propensity scores.

**
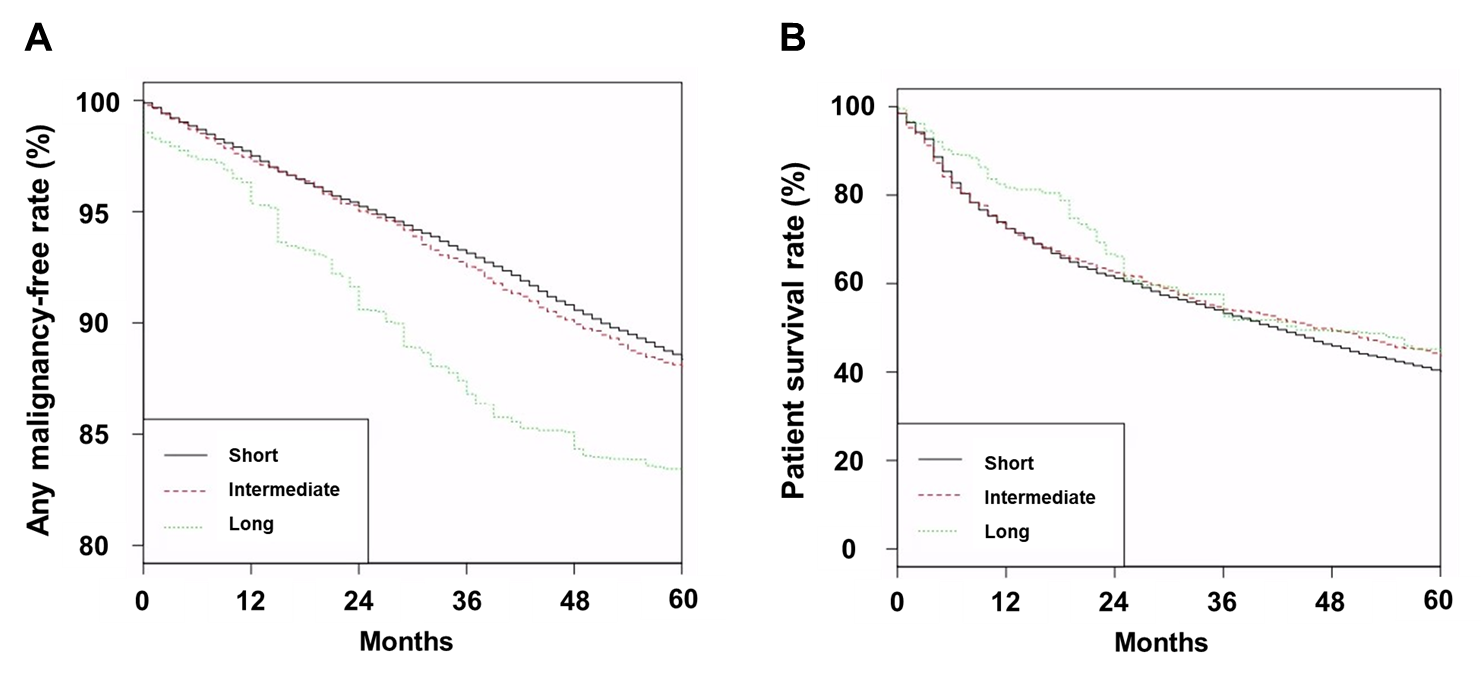
**

**Figure S3. Kaplan–Meier curves by type of erythropoiesis stimulating agents using balanced cohort.** (A) Any malignancy-free and (B) Patient survival rates.
